# Insights in the Ionic Conduction inside Nanoporous Metal-Organic Frameworks by Using an Appropriate Equivalent Circuit

**DOI:** 10.3390/ma14164352

**Published:** 2021-08-04

**Authors:** Abhinav Chandresh, Zejun Zhang, Lars Heinke

**Affiliations:** Institute of Functional Interfaces (IFG), Karlsruhe Institute of Technology (KIT), Hermann-von-Helmholtz-Platz 1, 76344 Eggenstein-Leopoldshafen, Germany; abhinav.chandresh@kit.edu (A.C.); zejun.zhang@partner.kit.edu (Z.Z.)

**Keywords:** metal-organic frameworks, proton conduction, ionic liquids, impedance spectroscopy

## Abstract

The conduction of protons and other ions in nanoporous materials, such as metal-organic frameworks (MOFs), is intensively explored with the aim of enhancing the performance of energy-related electrochemical systems. The ionic conductivity, as a key property of the material, is typically determined by using electrochemical impedance spectroscopy (EIS) in connection with a suitable equivalent circuit. Often, equivalent circuits are used where the physical meaning of each component is debatable. Here, we present an equivalent circuit for the ionic conduction of electrolytes in nanoporous, nonconducting materials between inert and impermeable electrodes without faradaic electrode reactions. We show the equivalent circuit perfectly describes the impedance spectra measured for the ion conduction in MOFs in the form of powders pressed into pellets as well as for MOF thin films. This is demonstrated for the ionic conduction of an aprotic ionic liquid, and of various protic solvents in different MOF structures. Due to the clear physical meaning of each element of the equivalent circuit, further insights into the electrical double layer forming at the MOF-electrode interface can be obtained. As a result, EIS combined with the appropriate reference circuit allows us to make statements of the quality of the MOF-substrate interface of different MOF-film samples.

## 1. Introduction

Electrochemical processes are key to many energy-related applications, such as fuel cells and batteries [1,2,3,4]. In many of these applications, the performance can be significantly improved by using advanced nanoporous materials [5]. In particular, metal-organic frameworks (MOFs) and covalent-organic frameworks (COFs), but also conventional nanoporous crystalline materials like zeolites have the potential to boost most electrochemical applications [5,6,7,8,9]. MOFs are solid materials, self-assembled from inorganic metal nodes connected by organic linker molecules. The application of MOFs in modern electrochemical applications, for instance in fuel cells, batteries, supercapacitors, and sensors has attracted significant attention [7,10,11,12,13]. For utilizing nanoporous materials, the conductivity of the material is among the key properties and the mobility of charges (e.g., protons, lithium, and other ions) is crucial. The material conductivity is typically experimentally explored by electrochemical impedance spectroscopy (EIS) [14,15]. In EIS, the current in an electrochemical cell is measured as a response to a small AC voltage (typically in the order of 10–300 mV) over a wide frequency range (typically in the order of 1 Hz to 1 MHz). For the data analysis, the correct equivalent circuit reflecting the electrochemical properties of the setup and sample need to be utilized. In various studies exploring the ionic conduction in MOFs, the authors use different equivalent circuits where the focus is on an *RC*-circuit (i.e., an ohmic resistance *R* in parallel combination with a capacitor *C*) [7,16,17,18,19,20,21,22,23]. In the complex plane plot of the impedance, also referred to as Nyquist plots, the *RC*-element gives a semi-circle with *R* as diameter. This diameter is typically used to calculate the conductivity of the material. Often, in addition to the high-frequency semi-circle, a low-frequency ray or spike is also observed. In many studies [20,24,25,26,27,28,29], the experimental impedance data are then described by an equivalent circuit similar to the Randles circuit, where the Nyquist plot shows a semi-circle at high frequencies with a diameter of an ohmic resistance connected to a low-frequency ray with an angle of 45°. The Randles equivalent circuit is composed of an ohmic resistance in serial with a Warburg (diffusion) element in parallel combination with a capacitance, i.e., it is an *RC*-element with a Warburg resistance in serial to *R* [14]. The Randles equivalent circuit was introduced for faradaic electrode reactions with diffusing components [14,30]. However, when using EIS to explore the ionic conduction in nanoporous materials like MOFs, the applied AC voltage is usually in the range of 10 mV to 300 mV, which is commonly smaller than the reduction or oxidation potentials of the explored materials and guest molecules. Thus, no faradaic electrode reaction takes place under typical experimental conditions. Therefore, the physical meaning of each element in the Randles equivalent circuit is questionable when applied to ionic conduction in MOFs. (In other words, the main problem is that there is typically no charge transfer across the MOF-electrode interface, i.e., faradaic reactions do not take place, being fundamental for the Randles circuit). Although the ohmic resistance seems to be correctly determined via the high-frequency semi-circle, the application of the Randles circuit to describe the ionic conduction in nanoporous materials is debatable.

For various ionic conductor systems, appropriate equivalent circuits have been discussed and justified [31,32,33,34]. Appropriate equivalent circuits where each component has a clear physical meaning have also been introduced for various nanoporous systems. Examples are MOF films with (low) electronic conductivity on substrates with interdigitated gold electrodes (IDEs) [35], zeolite-Cr_2_O_3_ films on IDEs used as gas sensors [36,37], zeolites embedded in polymer membranes [38], zeolite films as coatings on alloys [39], or for the film growth of a zeolite membrane [40]. To the best of our knowledge, a critical discussion of an equivalent circuit for the ionic conduction in nanoporous non-conducting materials, like (most) MOFs between two inert electrodes like gold is missing.

The aim of the present work is to introduce an appropriate equivalent circuit for the ionic conduction of electrolytes in the pores of nanoporous non-conducting host materials, where each element of the equivalent circuit has a physical meaning. We suggest and justify an equivalent circuit composed of the ohmic resistance of the material in serial with a constant phase element (CPE), reflecting the real capacitor of the double-layer at the electrode surfaces, in parallel combination with a CPE, reflecting the geometric capacity of the electrodes. We show that the equivalent circuit describes the ion conduction in nanoporous MOFs, for the material in the form of thin films grown on substrates with deposited electrodes (like IDEs), as well as in the form of MOF powders pressed into pellets sandwiched between two gold electrodes. The analysis is applied to the conduction of ionic liquids (ILs) in MOFs of type HKUST-1 (Hong Kong University of Science and Technology-1 [41]) in the form of thin films and in the form of MOF pellets as well as to the proton conduction of various molecules in pillared-layered MOF thin films. In these conduction processes, no faradaic reactions take place under the experimental conditions. We show that by using the appropriate equivalent circuit, further parameters in addition to the resistance (and correlated conductivity) of the material can be evaluated. We compare three MOF films which are made of the same structure in the same way, however, the substrate was functionalized in different ways, i.e., with two different self-assembled monolayers, and by UV-ozone treatment. We show that although the crystallinity and conductivity of the material are very similar, the EIS with the introduced equivalent circuit allows us to make qualitative statements on the MOF-substrate interface, which is commonly difficult to explore.

## 2. Materials and Methods

Surface-mounted MOF (SURMOF) thin films were prepared in a layer-by-layer (lbl) fashion on the substrates [42]. In detail, the lbl growth process consists of alternately exposing the substrate to the ethanolic solutions of the building units; that is, the metal nodes (here: 1 mM copper acetate) and the organic linkers (here: 0.2 mM benzene-1,3,5-tricarboxylic acid, BTC). Between each step, the sample surfaces were cleaned with pure ethanol. The SURMOF samples were prepared in 50 lbl synthesis cycles by using a spray method [43]. The substrates are glass sheets with deposited interdigitated gold electrodes (IDEs), obtained from DropSens (Metrohm DropSens, Oviedo, Spain). The total length of the gap between the gold electrodes is 1.69 m (=2 times 125 gaps of 6.76 mm length) and the gap width is 10 µm. Before the SURMOF synthesis, the substrates were treated in three different ways: (1) by UV-ozone treatment for 15 min to remove impurities and to increase the number of OH functional groups; (2) an OH-terminated self-assembled monolayer (SAM) was formed on the substrate by immersion in a thiol solution, this means a 11-mercapto-1-undecanol (MUD) SAM was formed on the Au electrodes upon immersion in 20 µm MUD ethanol solution for 24 h; and (3) a COOH-terminated SAM was formed upon immersion of the substrate in 20 µM MHDA (16-mercaptohexadecanoic acid) ethanol solution for 48 h. The samples were loaded with ionic liquid of type 1-butyl-3-methylimidazoliumbis (trifluormethylsulfonyl)imid, referred to as [BMIM][TFSI], by immersion for 20 min, as described in [27]. Upon IL loading, excess IL on the SURMOF surface was rinsed off with pure acetonitrile. The samples were placed in a homemade Teflon cell and the IDEs were contacted in a two-probe way. All EIS measurements were performed in an atmosphere of pure nitrogen.

HKUST-1 MOF in the form of crystalline powder was prepared by following standard synthesis methods [44]. For the loading of the MOF powder with IL, different amounts of [BMIM][TFSI] were added to the activated HKUST-1 powder separately, homogeneously mixed, and heated under a vacuum at 120 °C overnight. For the preparation of the IL@MOF-pellets, we followed standard directions [45] (See Appendix A for experimental details). The resulting pellets had cylindrical shapes of 13 mm diameter with a thickness of approximately 1.7–2.0 mm. The conduction of the pellets was measured by positioning the IL@MOF-pellets between 2 planar electrodes, which are gold thin film (150 nm thickness) on conducting Si-wafer.

In the manuscript, we also applied the suggested equivalent circuit to data previously published by our group. In detail, the data are IL of type [BMIM][TFSI] in SURMOFs of type HKUST-1 from [27], as well as butanediol and triazol in SURMOFs with a pillared-layer MOF (pl-MOF) structure of type Cu_2_(F_2_AzoBDC)_2_(dabco) from [26]. F_2_AzoBDC refers to (*E*)-2-((2,6-difluorophenyl)diazenyl)terephthalic acid and dabco to 1,4-diazabicyclo [2.2.2]octan. There, the SURMOFs were grown on glass substrates with deposited IDEs.

The X-ray diffractograms (XRD) were measured in an out-of-plane geometry using a Bruker D8-Advance diffractometer, equipped with a position-sensitive detector in θ–2θ geometry. A Cu-anode with a wavelength of *λ* = 0.154 nm was used. 

The electrochemical impedance spectra were measured with a Zurich Instruments MFIA Impedance Analyzer in a frequency range of 5 MHz to 0.5 Hz. The AC voltage was set to 300 mV. No DC voltage was applied.

All experiments were performed at room temperature (~295 K).

## 3. Results and Discussion

### 3.1. Introduction of Equivalent Circuit

**Figure 1:**
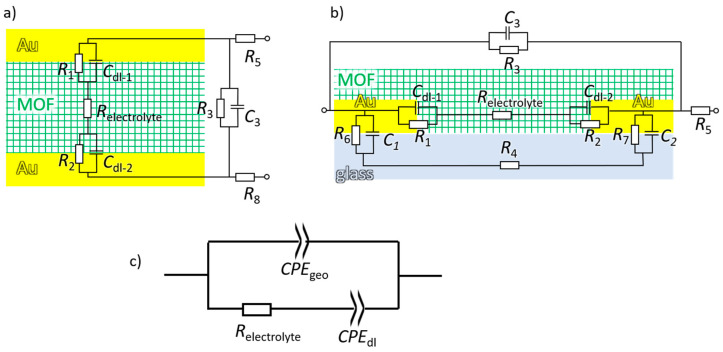
Sketch of a MOF-pellet between Au-electrodes (**a**), and of a MOF film on a glass substrate with deposited gold electrodes (**b**) with idealized equivalent circuits. The gold electrodes are sketched in yellow, the MOF (filled with electrolyte) in green, and the glass substrate in light blue. The parameters are: *R*_1_ and *R*_2_ are the charge transfer resistance between the electrodes and the electrolyte. *C*_dl-1_ and *C*_dl-2_ are the capacitances of the electrical double layers forming at the electrodes. *R*_electrolyte_ is the ohmic resistance of the electrolyte in the (MOF) material. *R*_5_ and *R*_8_ are the resistances of the setup, connections, and cables, potentially including some induction parts. *C*_3_ is the capacitance between the two electrodes (and also from other parts of the setup) and *R*_3_ is the resistance of potential leakage. In panel b, *R*_6_ and *R*_7_ are the charge transfer resistance from the electrode to the glass substrate, and *C*_1_ and *C*_2_ are the capacitances at these interfaces. *R*_4_ is the resistance of the glass substrate. (**c**) A simplified equivalent circuit for the ionic conduction in MOFs between two gold electrodes: the ohmic resistance of the electrolyte *R*_electrolyte_ in serial with a constant phase element of the double layer *CPE*_dl_ in parallel combination with a constant phase element of the geometric capacitance *CPE*_geo_. This circuit is referred to as (*R-CPE_dl_*)‖*CPE_geo_* circuit.

A sketch of the MOF pellet sandwiched between two planar electrodes is shown in Figure 1a. The top and bottom gold electrodes are contacted by wires with the impedance spectrometer, applying the AC voltage and measuring the impedance and phase. The sample is typically pressed in the form of a coin-shaped pellet. A sketch of the MOF material in the form of a film grown on nonconducting substrates with deposited electrodes is pictured in Figure 1b. Typically, the electrodes are interdigitated so that many gaps are connected in parallel (often many hundreds of gaps) with a total gap length in the range of 1 cm to 1 m. Please note, the size ratios in Figure 1a and 1b are not correctly pictured. The MOF pellets have a thickness typically in the range of 1 mm or less, and the planar top and bottom electrodes typically have a diameter in the range of 1 cm. For the MOF film on an IDE, the distance between the electrodes is typically in the range of 10–100 µm, and the thickness of the MOF film is usually 0.1–0.5 µm. The thickness of the gold IDEs is typically in the range of 0.1 µm. Thus, the *E*-field is essentially homogenous in the sample between the electrodes [46]. 

For both setups, the idealized electrical equivalent circuits are also shown in Figure 1a,b. Here, we focus on the ionic conduction of electrolytes in the pores of essentially insulating MOFs. The charge carriers of the electrolytes are ions (protons, cations, or anions), not electrons, and no faradaic reactions take place at the electrodes. The applied voltages are too small to cause reduction or oxidation reactions at the electrodes. On the other hand, the charge carriers in the gold electrodes are electrons and the electrodes are impermeable to the ions. Thus, the charge transfer resistances at the gold-electrolyte interface (i.e., the electrode-electrolyte interface, *R*_1_, and *R*_2_, see Figure 1) are very large (essentially infinity) and these paths do not contribute to the charge transfer. The leakage currents are typically very small (i.e., the resistances *R*_3_ and *R*_4_ are very large) and these paths can also be neglected. In the experimental setups, the resistance of the wires and connections (*R*_5_ and *R*_6_, eventually with a small inductance part) are very small and can typically be ignored. As a result, the equivalent circuit can be simplified to the ohmic resistance of the electrolyte (*R*_electrolyte_) in serial with a capacitor of the electrical double-layer at the electrode surfaces (combination of *C*_dl-1_ and *C*_dl-2_) in parallel combination with a capacitor reflecting the geometric capacity of the electrodes (*C*_3_). This simplified equivalent circuit is correct for perfectly homogenous interfaces at defect-free, single-crystalline, and flat electrodes (or liquid electrodes). In real electrochemical cells, the capacitance of the double layer (dl) shows a frequency dispersion, and the impedance $Z=1/2\pi ifC_{\mathrm{dl}}$ becomes to $Z=1/\left({\left(2\pi if\right)}^{n_{\mathrm{dl}}}CPE_{\mathrm{dl}}\right)$ [14,15]. Here, *n*_dl_ is the constant phase exponent with 0 < *n*_dl_ ≤ 1, also referred to as frequency power, and $CPE_{\mathrm{dl}}$ is the parameter related to the capacitance (in ${F\;s}^{n_{\mathrm{dl}}-1}$), also referred to as effective capacitance. The main reasons for the frequency dependence are heterogeneities at the electrodes, causing dispersion of the relaxation times of the double-layer polarizations [47,48,49]. (See also chapter 8 in [14]) Usually, the heterogeneities of the double layer are caused by the poly-crystallinity and roughness of the electrodes [47,48,49]. Potential impurities and adsorbates also contribute to the dispersion of relaxation times [14]. Here, the MOF material may possess additional inhomogeneities, resulting in a more heterogeneous electrical double-layer. Moreover, for films grown on IDEs deposited on non-conducting substrates, the electrode interface is obviously heterogeneous since the electric field at the electrode surface is inhomogeneous [46]. Heterogeneities and roughness in the film morphology and deviations from the ideal geometry, cause a frequency dependence of the geometric capacitor [14,15]. As a result, both capacitors in the simplified equivalent circuit need to be substituted by CPEs. This results in the equivalent circuit shown in Figure 1c, referred to as (*R-CPE*_dl_)‖*CPE*_geo_-circuit.

The complex impedance *Z* of the equivalent circuit (Figure 1c) is given by

(1) $$\frac{1}{Z}=\frac{1}{R+2/\left({\left(2\pi if\right)}^{n_{\mathrm{dl}}}CPE_{\mathrm{dl}}\right)}+\frac{1}{1/\left({\left(2\pi if\right)}^{n_{\mathrm{geo}}}CPE_{\mathrm{geo}}\right)}.$$

*R* denotes the ohmic resistance of the material between the electrodes, *i* is the imaginary number (i.e., *i*^2^ = −1), *f* is the AC frequency, *CPE*_dl_ is the CPE capacitance of the double layer at one electrode and *CPE*_geo_ is the geometric CPE capacitance. The constant phase exponent of the double layer and geometric capacitances are given by *n*_dl_ and *n*_geo_, which are in the range between 1 and 0. Please note the factor 2 of the impedance of *CPE*_dl_, indicating the double layers at both electrodes. For simplicity, it is assumed that both electrodes are identical.

**Figure 2:**
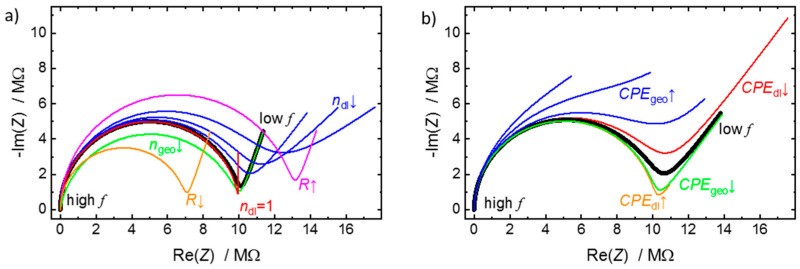
Nyquist plots for the (*R-CPE*_dl_)‖*CPE*_geo_ -circuit with typical parameters. (**a**) The black spectrum is calculated for *R* = 10 MΩ, *CPE*_dl_ = 1 × 10^−7^ ${\;F\;s}^{n_{\mathrm{dl}}-1}$, *n*_dl_ = 0.8, *CPE*_geo_ = 1 × 10^−10^ ${\;F\;s}^{n_{\mathrm{geo}}-1}$, *n*_geo_ = 1. The green line is for *n*_geo_ = 0.8, orange for *R* = 7 MΩ, magenta for *R* = 13 MΩ, red for *n*_dl_ = 1 and the blue lines are for *n*_dl_ = 0.6, 0.5 and 0.4 (left to right). The other parameters correspond to the parameters of the black line. (**b**) The black spectrum is calculated for *R* = 10 MΩ, *CPE*_dl_ = 1 × 10^−7^ ${\;F\;s}^{n_{\mathrm{dl}}-1}$, *n*_dl_ = 0.6, *CPE*_geo_ = 1 × 10^−10^ ${\;F\;s}^{n_{\mathrm{geo}}-1}$, *n*_geo_ = 1. The green line is for *CPE*_geo_ = 2 × 10^−11^ ${\;F\;s}^{n_{\mathrm{geo}}-1}$, orange for *CPE*_dl_ = 4 × 10^−7^ ${F\;s}^{n_{\mathrm{dl}}-1}$, red for for *CPE*_dl_ = 5 × 10^−8^ ${\;F\;s}^{n_{\mathrm{dl}}-1}$, and the blue lines are for *CPE*_geo_ = 1, 4 and 10 × 10^−9^ ${\;F\;s}^{n_{\mathrm{geo}}-1}$ (lower, middle, and upper line). The other parameters correspond to the parameters of the black line. The frequency range is from 1 Hz to 5 MHz. The high frequency range is close to zero impedance.

For a better understanding of the parameters, the effects of the individual parameters are discussed. The complex plane plots of the impedance, also referred to as Nyquist plots, are shown for typical values in Figure 2. In the Nyquist plots, the shape is a characteristic semi-circle in the high-frequency range and a straight line in the low-frequency range. For *n*_dl_ = 1 (red spectrum in Figure 2a), the low-frequency line is vertical in the Nyquist plot (usually at Re(*Z*) = *R*). With decreasing values of *n*_dl_, the low-frequency line becomes less steep and the branch becomes larger (i.e., the impedance of the *CPE*_dl_ element becomes larger). The high-frequency semi-circle has a diameter of *R*. For decreasing *n*_geo_, the semi-circle is flattened (green spectrum). It should be noted that for *n*_geo_ = 1 and *n*_dl_ = 0.5, the semi-circle is perfectly round, and the low-frequency spike has an angle of 45° in the Nyquist plot, just like the Randles circuit, however, the physical meaning of the components are different, see above.

By varying *CPE*_dl_ and *CPE*_geo_, the shapes of the impedance spectra in the Nyquist plot vary, see Figure 2b. For small *CPE*_geo_ and large *CPE*_dl_, the form of the semi-circle and the straight line at low frequencies is clearly visible, and at the local minimum of -Im(*Z*) the value of Re(*Z*) corresponds to *R*. For increasing *CPE*_geo_ and decreasing *CPE*_dl_, the local minimum of -Im(*Z*) increases, eventually vanishes, and the semi-circle and the low-frequency rays merge.

### 3.2. Application of the Equivalent Circuit to the Ionic Conduction in MOF Films and Pellets

**Figure 3:**
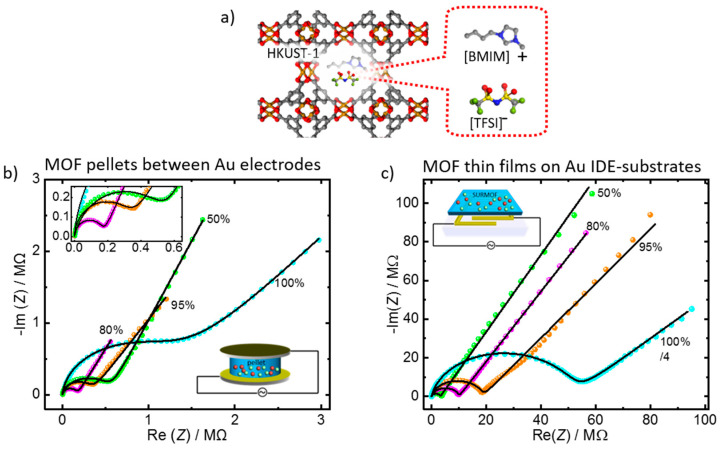
IL@HKUST-1. (**a**) Structure of IL of type [BMIM][TFSI] in HKUST-1 (**b**) and (**c**) Nyquist plots for the sample in the form of a MOF-pellet sandwiched between 2 gold electrodes (**b**), and in the form of a thin film on a glass substrate with interdigitated gold electrodes (**c**). The IL pore fillings are 50%, 80%, 95%, and 100%, as indicated. The black lines are the fits with the (*R-CPE*_dl_)‖*CPE*_geo_ circuit. The frequency range is 100 Hz to 5 MHz (**b**), and 0.5 Hz to 5 MHz (**c**). Please note, for better visibility, the impedance values for 100%-loading in panel (**c**) are divided by 4, to allow the presentation in the same plot. The SURMOF sample was 0.25 µm thick, deposited on a substrate with IDEs of 10 µm gap distance and a total gap length of 1.69 m. The data of panel (**c**) are taken from [27] with copyright permission from 2019 American Chemical Society.

The equivalent circuit is applied to the conduction of ionic liquids in MOF materials, in the form of pellets and thin films—Figure 3. In detail, MOFs of type HKUST-1 filled with various loadings of IL of type [BMIM][TFSI] are studied. Figure 3b shows the ionic conduction in MOFs in the form of pellets, and Figure 3c shows the ionic conduction in a MOF film grown on a substrate with deposited (IDE) electrodes. In both cases, the characteristic semi-circle at high frequencies and the straight line at low frequencies are clearly visible. The experimental data can be well described by the suggested equivalent circuit.

**Figure 4:**
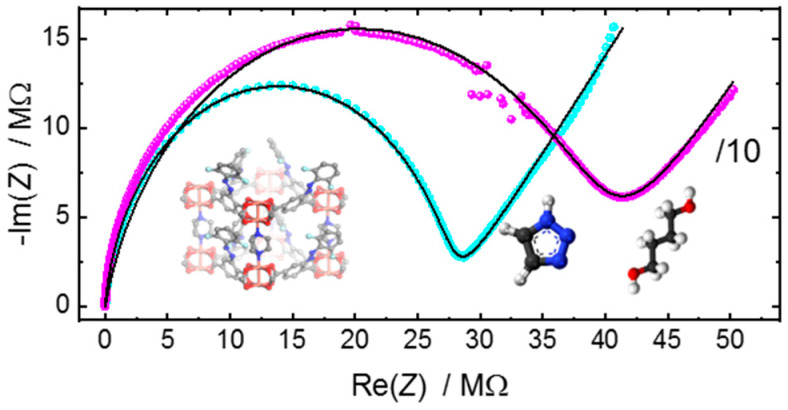
Nyquist plots of the proton-conduction of triazole (cyan) and butanediol (magenta) in a pillared-layered SURMOF on an IDE-substrate. The SURMOF structure and the molecules are sketched in the insets. The impedance values for butanediol are divided by 10, to allow the presentation in the same plot. The black lines are the fits with the (*R-CPE*_dl_)‖*CPE*_geo_. The frequency range is 0.5 Hz to 5 MHz. The SURMOF sample was 150 nm thick, deposited on a substrate with IDEs of 10 µm gap distance, with a total gap length of 2 cm. The frequency range is 0.5 Hz to 5 MHz. The data were previously published in [26] with copyright permission from 2018 WILEY-VCH Verlag GmbH & Co. KGaA.

For proton-conducting materials, there is also no charge transfer between the electrode and the electrolyte, i.e., through the electrode-electrolyte interface. Thus, the suggested equivalent circuit can also be applied. In Figure 4, the Nyquist plots measured for two different proton-conducting molecules, that are 1,2,3-triazole and 1,4-butanediol, in the pores of a MOF thin film are shown. The data can be described well with the (*R-CPE*_dl_)‖*CPE*_geo_ equivalent circuit.

**Figure A1:**
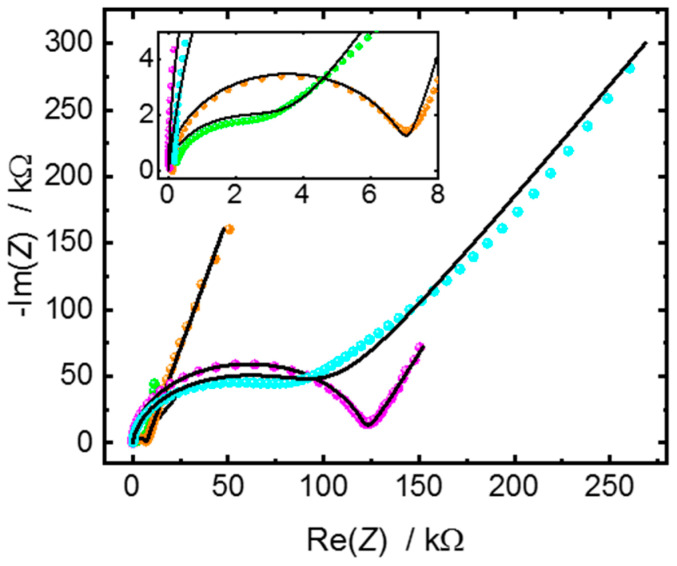
Nyquist plots of pure liquids of 1,2,3-triazole (cyan), 1,4-butanediol (magenta), deionized water (orange) and pure IL (green). Butanediol and water droplets were deposited on an IDE-substrates with 10 µm gap distance and a total gap length of 1.69 m. The area of each electrode is approximately 17 mm^2^. Triazole and IL droplets were deposited on electrodes with a gap of 10 µm gap distance and a gap length of 1 mm. The area of each electrode is 0.01 mm^2^. The frequency range is from 100 Hz (triazole), 10 Hz (butanediol), 10 Hz (water), and 1000 Hz (IL) to 5 MHz. The data are fitted with the (*R-CPE*_dl_)‖*CPE*_geo_ equivalent circuit, black lines, and the parameters are given in Table A1.

As a reference, the Nyquist plots of pure IL and of protic liquids, triazole, deionized water, and butanediol, on the substrates with the deposited electrodes are shown in the appendix, Figure A1. The qualitative shape of the Nyquist plot of the pure liquids and the liquids in the MOF confinement are similar.

**Figure A2:**
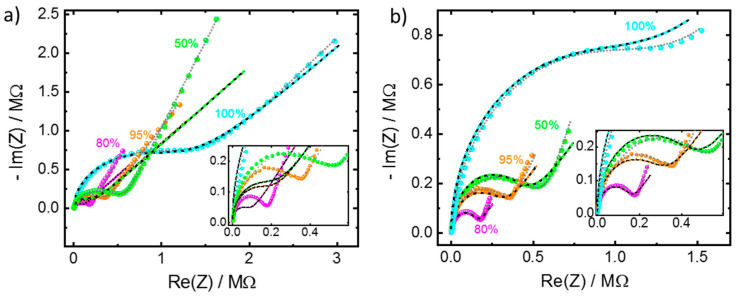
Nyquist plots for the IL@HKUST-1 in the form of a MOF-pellet sandwiched between 2 gold electrodes, see Figure 3b. The experimental data are shown as spheres, the grey-dotted lines are the fits with the (*R-CPE*_dl_)‖*CPE*_geo_ circuit (same data as in Figure 3b). The black-color-dashed lines are the best fits by Randles equivalent circuit. The IL pore fillings are 50%, 80%, 95%, and 100%, as indicated. The frequency range in panel (**a**) is 100 Hz to 5 MHz, and in panel (**b**) is 1109 Hz to 5 MHz. Only the plotted data are used for the fitting by the models. The major reason for the poor description of the experimental data by the Randles circuit is the fact that the Warburg resistance of the Randles circuit results in a low-frequency ray with an angle of 45° in the Nyquist plots, which is often not the case in the experimental data. This results in poor fits, especially when the low-frequency range is recorded, see panel (**a**).

**Figure A3:**
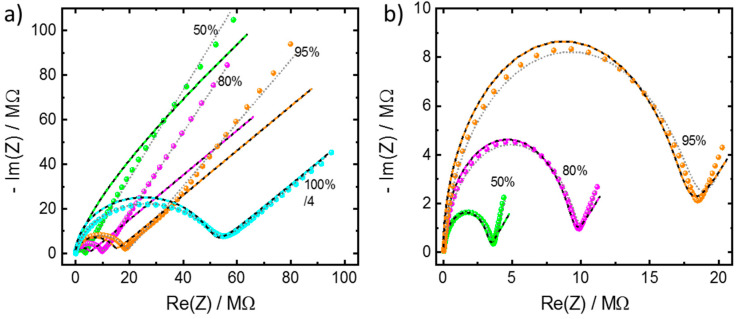
Nyquist plots for the IL@HKUST-1 in the form of a thin film on a glass substrate with interdigitated gold electrodes, see Figure 3c. The experimental data are shown as spheres, the grey-dotted lines are the fits with the (*R-CPE*_dl_)‖*CPE*_geo_ circuit (same data as in Figure 3c). The black-color-dashed lines are the best fits by Randles equivalent circuit. The IL pore fillings are 50%, 80%, 95%, and 100%, as indicated. The frequency range in panel (**a**) is 0.5 Hz to 5 MHz, and in panel (**b**) is 107 Hz to 5 MHz. Only the plotted data are used for the fitting by the models. Like in Figure A2, the major reason for the poor description of the experimental data by the Randles circuit is the fact that the Warburg resistance of the Randles circuit results in a low-frequency ray with an angle of 45° in the Nyquist plots, which is often not the case in the experimental data. This results in poor fits, especially when the EIS data at the low-frequency range is recorded—see panel (**a**).

**Figure A4:**
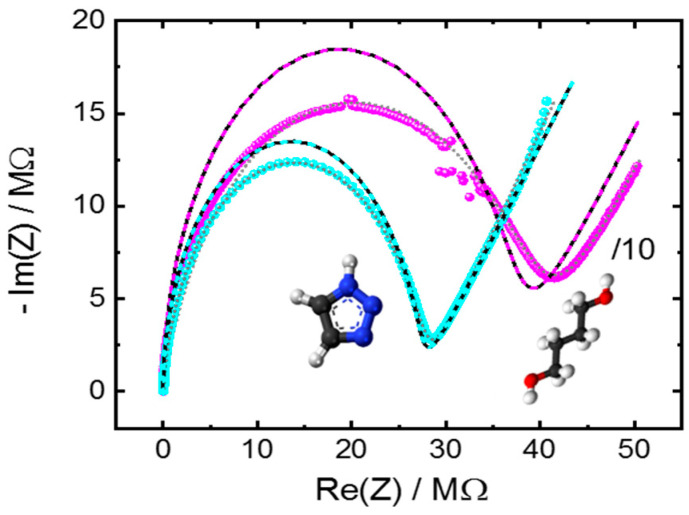
Nyquist plots of the proton-conduction of triazole (cyan) and butanediol (magenta) in a pillared-layered SURMOF on an IDE-substrate—see Figure 4. The experimental data are shown as spheres, the grey-dotted lines are the fits with the (*R-CPE*_dl_)‖*CPE*_geo_ circuit (same data as in Figure 4). The black-color-dashed lines are the best fits by Randles equivalent circuit. The frequency range is from 0.5 Hz to 5 MHz.

**Figure A5:**
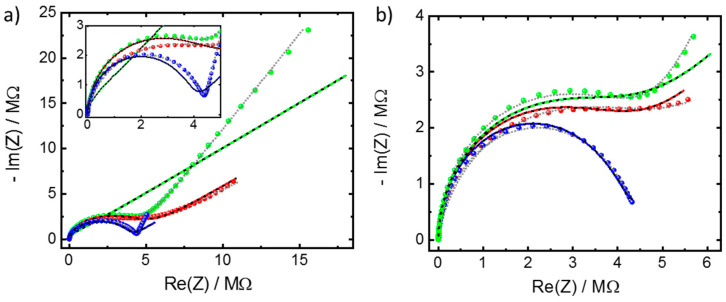
Nyquist plots of IL in HKUST-1 SURMOF films on substrate functionalized with MUD-SAM (green) MHDA-SAM (red) and with UV-ozone for 30 min (blue)—see Figure 5. The experimental data are shown as spheres, the grey-dotted lines are the fits with the (*R-CPE*_dl_)‖*CPE*_geo_. The black-color-dashed lines are the best fits by Randles equivalent circuit. The frequency range in panel (**a**) is 3.5 Hz to 5 MHz, and in panel (**b**) is 56 Hz to 5 MHz. Only the plotted data are used for the fitting by the models. Like in Figure A2 and Figure A3, the major reason for the poor description of the experimental data by the Randles circuit is the fact that the Warburg resistance of the Randles equivalent circuit results in a low-frequency ray with an angle of 45° in the Nyquist plots, which is often not the case in the experimental data. This results in poor fits, especially when the low-frequency range is recorded, see panel (**a**).

The parameters used to describe the experimental data in Figure 3 and Figure 4 with the (*R-CPE*_dl_)‖*CPE*_geo_ model are shown in Table 1. The constant phase exponents of the electrical double layer, *n*_dl_, is in all cases larger than 0.5. This indicates that a (purely phenomenological) description of the data by a Randles-cell-like circuit results in a larger deviation than by the (*R-CPE*_dl_)‖*CPE*_geo_ circuit. Indeed, in comparison to the fits with the Randles-cell-like circuit [26,27], the (*R-CPE*_dl_)‖*CPE*_geo_ circuit allows a significantly better reflection of the experimental data (see Appendix A, Figure A2, Figure A3, Figure A4 and Figure A5).

The *n*_dl_ values of the pure liquids are in the range of 0.66 and 0.84 (Table A1), while the *n*_dl_ values of the MOF samples are significantly smaller. The *n*_dl_ values of the MOF pellets are in the range of 0.55 to 0.73 and 0.51 and 0.71 for the SURMOFs on the IDE-substrates. The smaller constant phase exponents indicate that the interfaces with the MOF are more heterogeneous than the interfaces of the pure liquids.

EIS is typically applied to nanoporous materials with the objective of determining the material’s conductivity σ, which is calculated from the determined ohmic resistance *R* of the sample. Usually, the equation σ = *d A*^−1^ *R*^−1^ is used, where *d* is the distance between the electrodes and *A* is the cross-sectional area of the sample. For MOF films on IDE-substrates, *A* is the total gap length times the average MOF film thickness. For MOF-pellets, *A* is the area of the pellet top or bottom surface. The calculated conductivities for the examples in Figure 3 and Figure 4 are given in Table 1. The conductivity values determined by the suggested equivalent circuit match the conductivity values previously determined [26,27]. The deviations are only a few percent.

### 3.3. Insights in the MOF-Electrode Interface 

**Figure 5:**
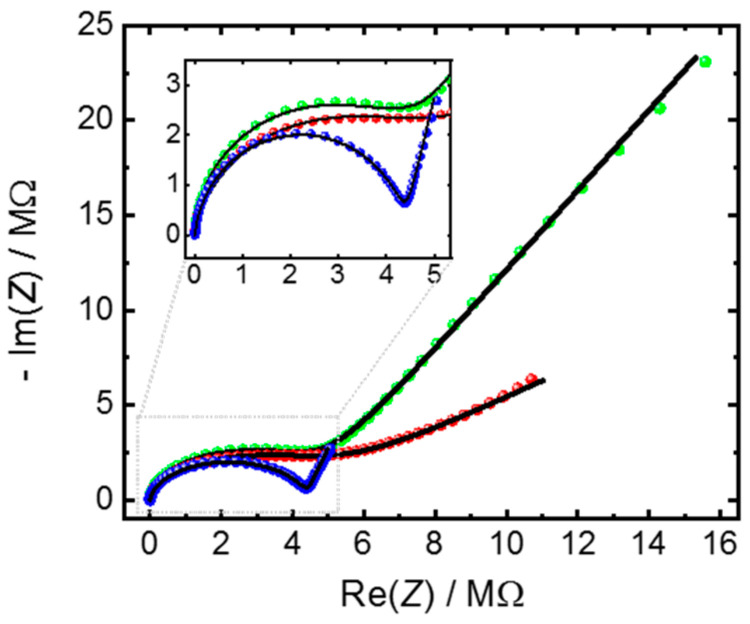
Nyquist plots of IL [BMIM][TFSI] in HKUST-1 SURMOF films. The SURMOF films are grown on the IDE substrates, whose surface has been treated in different ways before the SURMOF synthesis: One substrate has an OH-terminated MUD-SAM (green), one has a COOH-terminated MHDA-SAM (red) and one substrate was exposed to UV-ozone for 30 min before the SURMOF synthesis (blue). The black lines are the fits with the (*R-CPE*_dl_)‖*CPE*_geo_ circuit, where the parameters are shown in Table 2. The substrates are glass sheets with IDEs of 10 µm gap distance and a total gap length of 1.69 m. The area of each electrode is approximately 17 mm^2^. The frequency range is 3.5 Hz to 5 MHz.

**Figure A6:**
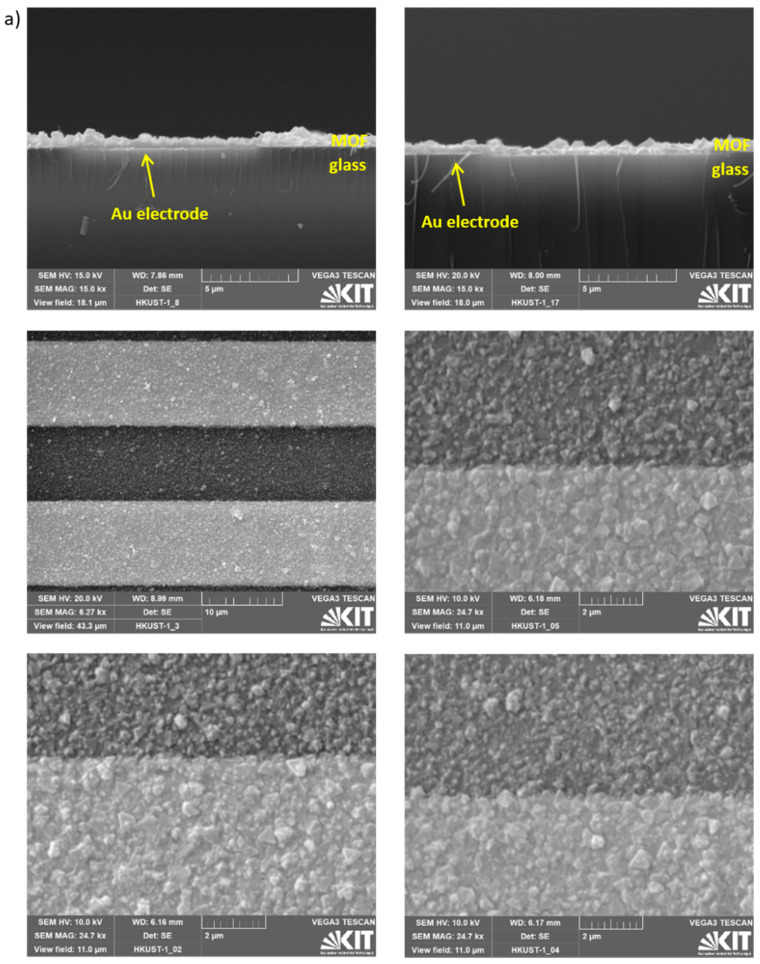

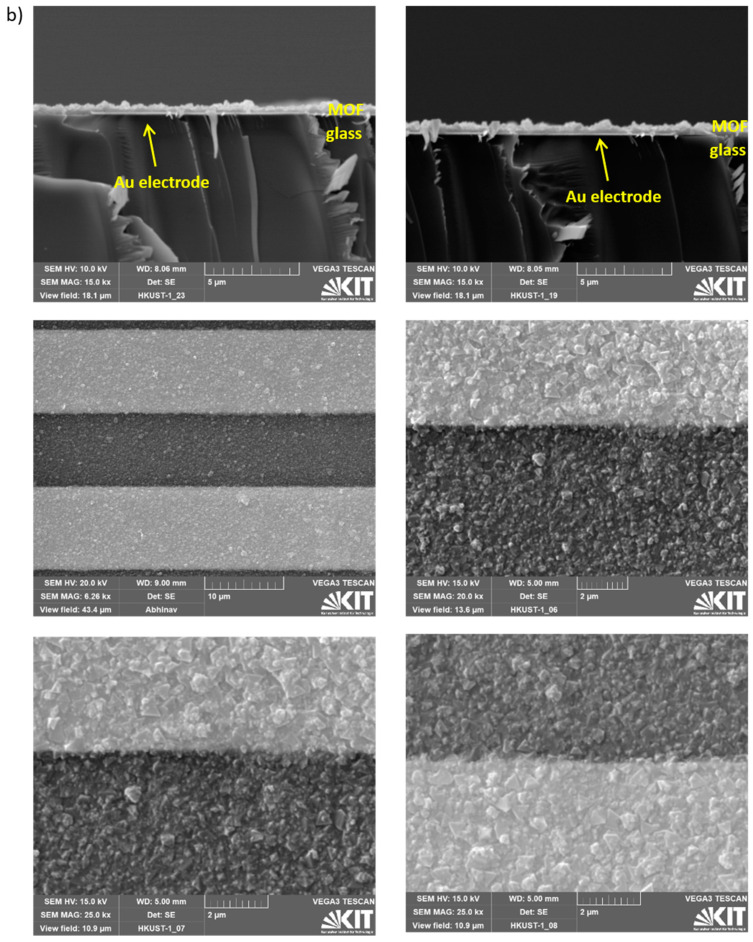

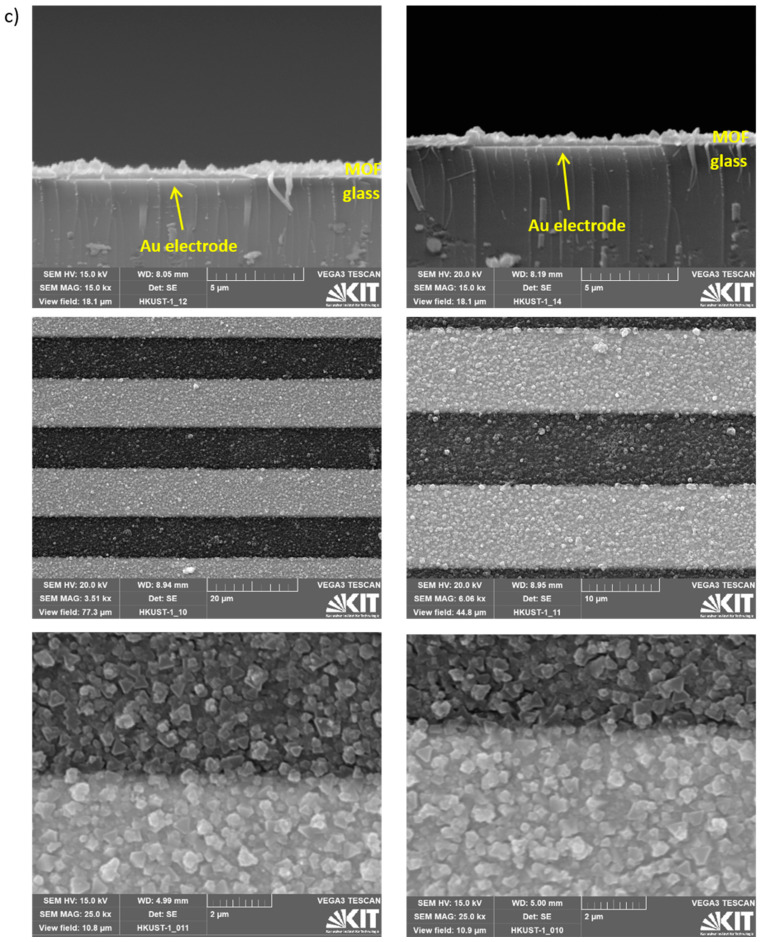
SEM images of the samples used in Figure 5. Cross-sections of the broken samples and top views of the samples are shown. The samples are HKUST-1 SURMOFs, grown on the IDE-substrates upon functionalization with MHDA (**a**), with MUD (**b**), and by UV-ozone treatment (**c**). The thicknesses of all 3 samples are 0.5 ± 0.1 µm, determined from the cross-section pictures.

**Figure A7:**
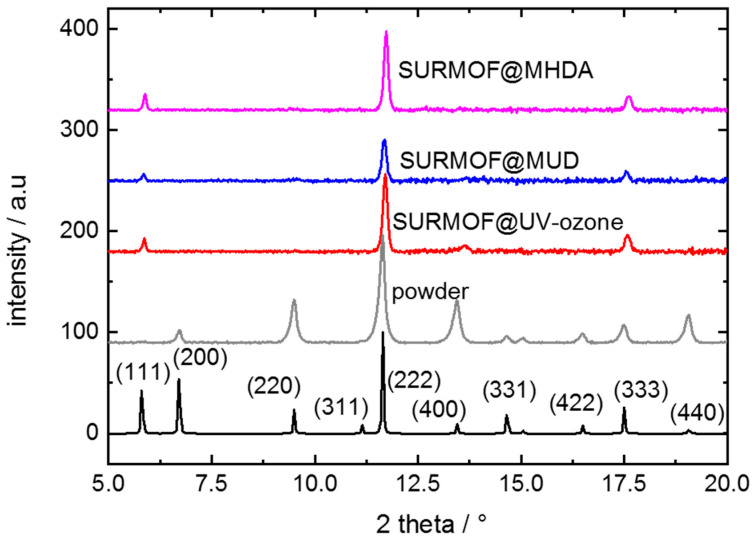
Out-of-plane XRD of HKUST-1 samples. The XRD of the MOF powder (for Figure 3b) is shown in grey. The XRDs of the SURMOFs grown on substrates with MHDA, MUD, and UV-ozone functionalization (see labels, for Figure 5) are shown in magenta, blue, and red, respectively. The black line is the calculated diffractogram with the labelled diffraction peaks. While the MOF powder shows all diffraction peaks, the SURMOF samples are grown in (111) orientation, showing only the (*nnn*) XRD peaks.

In addition to the quantification of the conductivity, the appropriate equivalent circuit can provide further details of the electrochemical system. We use the method to compare the properties of very similar SURMOF samples of the HKUST-1 structure. The samples are prepared on the same kind of IDE-substrates, but the substrate treatment, before the SURMOF synthesis, varies. To this end, three popular methods for functionalizing the substrate surface prior to the SURMOF-lbl-synthesis were used: (1) one substrate was treated with UV-ozone [50,51,52]; (2) one substrate was functionalized with -OH-terminated MUD-SAM [52,53,54]; and (3) one substrate was functionalized with -COOH-terminated MHDA-SAM [55,56]. The SURMOF films are prepared simultaneously with the same methods, so the MOF film thickness is essentially the same (see also the scanning electron images, Figure A6). The crystallinities of all samples are also comparable—Figure A7. In Figure 5, the Nyquist plots of the three SURMOF samples filled with IL are shown. All Nyquist plots can be described with the (*R-CPE*_dl_)‖*CPE*_geo_-reference-circuit, and the parameters are given in Table 2. The ohmic resistances of all three samples are in the range of 4.4–4.9 MΩ, corresponding to a conductivity of approximately 2.5 µS m^−1^. However, a surprising feature is that the *CPE*_dl_-parameter varies significantly, which can be seen by the different size of the low-frequency spike.

The capacitance of a CPE can be calculated by $C_{\mathrm{dl}}={\left(R\times CPE_{\mathrm{dl}}\right)}^{1/n_{\mathrm{dl}}}/R$ (see ref. [57,58], or chapter 8 in ref. [14])—Table 2. While the SURMOF-sample prepared on the substrate functionalized via UV-ozone treatment shows a large dl-capacitance, the dl-capacitance of the sample on the COOH-functionalized substrate is smaller, and the smallest dl-capacitance has the sample on the OH-functionalized substrate. Due to the inhomogeneity of the IDE and the electric field at the electrode-MOF interface, the precise determination of the dl-thickness is clearly not possible. Nevertheless, we use the dl-capacitance for a comparison of the three samples. The capacitance of the electrical double layer is given by $C=\varepsilon_{r}\varepsilon_{0}A/\lambda$, where $\varepsilon_{r}$ is the dielectric constant of the electrolyte, $\varepsilon_{0}$ is the vacuum permittivity ($\varepsilon_{0}=8.854\times 10^{-12}\;{F\;m}^{-1}$), and λ is the thickness of the electrical double layer. *A* is the area of the double layer (i.e., the contact area between the electrode and electrolyte), which is difficult to assess, although the total electrode areas are known. Here, λ and $\varepsilon_{r}$ are average values and the total area of the electrode is used for *A*. Since the geometries of the electrodes, the MOF structure, and the electrolyte of the three samples are identical, the comparison of the estimated dl-thicknesses is possible. It shows that IL in the MOF pores of the UV-ozone treated sample has the smallest dl-thickness, followed by the IL in the MOF on the COOH-terminated SAM substrate, and the OH-terminated SAM substrate. In all these MOF samples, the determined dl-thicknesses are much larger than the dl-thickness determined of pure IL on the Au electrodes—see Table 2 and Table A1, as well as for literature reference values [59,60,61]. We believe that the large dl-thickness of the IL in the MOF confinement is caused by structural defects and deficiency at the MOF-electrode interface. We speculate that defective MOF regions which are impermeable for the IL are present at the electrode interface cause large distances between the IL-electrolyte and the electrode, and, thus, causing large assessed dl-thicknesses. These defective regions are presumably dense copper acetate precursor species, as observed by atomic force microscopy and infrared spectroscopy [62]. In addition, the defective regions decrease the contact area *A* of the electrolyte-electrode interface. Since *A* was set constant for calculating the dl-thickness, the calculated dl-thickness is smaller. Regardless of the nature of the defects, we can use the calculated dl-thickness to classify the quality of the MOF samples. The sample where the IL has the smallest dl-thickness has the highest porosity at the interface, and, thus, the highest quality. As result, the interface of the MOF film made on the substrate upon UV-ozone treatments has the highest quality.

It should be noted that calculating the dl capacity for the IL-filled MOF pellets shown in Figure 3b results in values of about 1 nF, and in dl-thicknesses of about 1 µm. We believe this large calculated dl-thickness is not caused by defective MOF regions, rather than by the small contact area between the IL@MOF-pellet and the gold electrodes. Better contact with a larger contact area would increase the dl-capacitance, resulting in a more pronounced semi-circle—see Figure 2b.

## 4. Conclusions

The conduction of ions in nonconducting nanoporous MOF materials was explored. For the material in the form of pellets sandwiched between two inert, nonpermeable gold electrodes as well as for thin films grown on nonconducting substrates with two deposited electrodes (such as interdigitated electrodes), the equivalent circuit is derived, referred to as (*R-CPE*_dl_)‖*CPE*_geo_. The application of the equivalent circuit allows the description of the experimentally determined impedance data, significantly more precisely than with the previously used reference circuits. Moreover, each component has a clear physical meaning and can be correlated to the electrochemical system. In addition to the determination of the material conductivity, the application of the equivalent circuits provides further insights, showing that the double-layer of the electrolyte in the MOF material is significantly more heterogeneous than the double-layer of the pure electrolyte. The IL@MOF-electrode interface can be qualitatively characterized by comparing the assessed double-layer-thicknesses. The analysis shows severe differences in the interface properties although the conduction properties are similar.

Although the interface of the nanoporous film and the substrate is of paramount importance for the application, e.g., in batteries, supercapacitors, and electrocatalysis, their direct exploration by advanced methods, such as high-resolution microscopy, presents a severe challenge. Thus, we believe the presented method will help to explore the MOF-support interface and will contribute to enhanced MOF-device performances.

## Figures and Tables

**Table 1:** The parameters determined for the examples shown in Figure 3 and Figure 4. IL refers to [BMIM][TFSI] ionic liquid. The percentage gives the IL pore filling. pl-MOF stands for pillared-layer SURMOF; here, it is Cu_2_(F_2_AzoBDC)_2_(dabco).

Form	Electrolyte	MOF Host	*R*/MΩ	${CPE}_{\mathrm{dl}}/10^{-9}\;\;F\;\;s^{n_{\mathrm{dl}}-1}$	*n*_dl_	${CPE}_{\mathrm{geo}}/10^{-12}\;F\;\;s^{n_{geo}-1}$	*n*_geo_	Conductivity *σ*/µS m^−1^
MOF pellets between Au-electrodes	IL 100%	HKUST-1	1.29	20.4	0.55	25.1	0.95	5.8
	IL 95%	HKUST-1	0.34	19.9	0.64	12.0	0.98	22.2
	IL 80%	HKUST-1	0.18	25	0.70	18.0	0.96	43.1
	IL 50%	HKUST-1	0.57	6.36	0.73	34.7	0.84	13.2
MOF thin film on IDE-substrate	IL 100%	HKUST-1	205	4.58	0.51	1.91	0.90	0.12
	IL 95%	HKUST-1	18.7	9.14	0.61	1.91	0.89	1.3
	IL 80%	HKUST-1	9.98	9.56	0.68	1.83	0.90	2.4
	IL 50%	HKUST-1	3.21	7.58	0.71	1.85	0.91	7.4
	butanediol	pl-MOF	393	6.64	0.52	8.878	0.84	8.5
	triazole	pl-MOF	27.3	52	0.53	9.9	0.93	122

**Table 2:** The parameters determined from the EIS data in Figure 5. The HKUST-1 SURMOFs are prepared on the same kind of IDE-substrates but with different substrate functionalizations. The electrolyte is [BMIM][TFSI] ionic liquid with 50% pore filling. The thickness of the double layer is assessed by $\lambda_{\mathrm{dl}}=\varepsilon_{r}\varepsilon_{0}A_{\mathrm{electrode}}/C_{\mathrm{dl}}$. A value of 2.8 was used for the permittivity, $\varepsilon_{r}$, which is the value for the empty HKUST-1 MOF [63]. Please note, the permittivity value of the MOF is actually somewhat larger, due to the IL pore fillings, increasing the calculated thickness of the double layer.

Substrate Functionalization	*R*/MΩ	*CPE*_dl_ $/10^{-9}\;\;F\;\;s^{n_{\mathrm{dl}}-1}$	*n*_dl_	*CPE*_geo_ $/10^{-12}\;F\;\;s^{n_{geo}-1}$	*n*_geo_	Conductivity *σ*/µS m^−1^	Double Layer Capacitance *C*_dl_/nF	Estimated Double Layer Thickness λ_dl_/nm ^#^
UV-ozone	4.40	51.9	0.85	99.2	0.94	2.7	40.2	10.4
MUD SAM	4.87	8.2	0.71	53.0	0.99	2.4	2.3	179.9
MHDA SAM	4.57	51.0	0.46	154	0.89	2.6	9.6	43.5

^#^ The estimation of the double layer thickness requires a homogenous double-layer, which is not given in the present case. Since all 3 samples are prepared on the same kind of substrates, the calculated numbers can be compared but their absolute values are debatable.

## Data Availability

All data generated or analyzed during this study are either already published (as indicated) or are available from the corresponding author on reasonable request.

## Appendix A

### Appendix A.1. MOF Powder Synthesis, IL Loading and Pellet Preparation

HKUST-1 MOF in the form of crystalline powder was prepared by following standard synthesis methods [44]. In detail, Cu(NO_3_)_2_·3H_2_O (0.87 g, 3.6 mmol) and BTC (0.42 g, 2.0 mmol) were dissolved in 24 mL of a mixture of ethanol and deionized water (1:1 *v*/*v*). The solution was kept in a Teflon-lined autoclave at 100 °C for 24 h. Upon cooling to room temperature, a crystalline powder was obtained. The powder was centrifuged and washed thoroughly with a mixture of water and ethanol (1:1 *v*/*v*). The powder was activated under a vacuum at 120 °C for 16 h. For the loading of the MOF powder with IL, different amounts of [BMIM][TFSI] were added to the activated HKUST-1 powder separately, homogeneously mixed and heated under a vacuum at 120 °C overnight. In detail, the IL amount was 74.9 mg of IL per 100 mg MOF for 100% loading, 71.1 mg of IL per 100 mg MOF for 95% loading, 59.9 mg of IL per 100 mg MOF for 80% loading, 37.4 mg of IL per 100 mg MOF for 50% loading, and 11.2 mg of IL per 100 mg MOF for 15% loading. The percentage of the pore filling was previously determined [27]. For the preparation of the IL@MOF-pellets, we followed standard directions [45]. In detail, for each pellet, 200–400 mg of the material were placed inside a stainless steel press and the pellet was formed under a pressure of approximately 150 MPa. The resulting pellets had cylindrical shapes of 13 mm diameter with a thickness of approximately 1.7–2.0 mm. The conduction of the pellets was measured by positioning the IL@MOF-pellets between 2 planar electrodes, which are gold thin film (150 nm thickness) on conducting Si-wafer.

### Appendix A.2. Additional Data—Nyquist Plots, Determined Parameters, SEM Images and XRD

**Table A1:** The parameters determined for the impedance data in Figure A1. IL refers to [BMIM][TFSI] ionic liquid. The thickness of the double layer is assessed by $\lambda_{dl}=\varepsilon_{r}\varepsilon_{0}A_{\mathrm{electrode}}/C_{\mathrm{dl}}$. For the permittivity $\varepsilon_{r}$, the following values were used: 14 for IL [64], 40 for triazole [65], 31.9 for butanediol [66], and 80 for water.

Electrolyte	*R*/kΩ	*CPE*_dl_ $/10^{-9}\;F\;\;s^{n_{\mathrm{dl}}-1}$	*n*_dl_	*CPE*_geo_ $/10^{-12}\;F\;\;s^{n_{geo}-1}$	*n*_geo_	Double Layer Capacitance *C*_dl_/nF	Estimated Double Layer Thickness λ_dl_/nm
IL	3.7	68	0.67	179	0.95	1.15	1
butanediol	121	1242	0.73	270	0.99	609	7.8
triazole	101	82	0.66	168	0.94	6.94	0.5
water	7.1	353	0.84	248	1	113	105
